# Current status and advances in comprehensive treatment of acute ischemic stroke in China: from evidence-based guidelines to clinical practice

**DOI:** 10.3389/fneur.2026.1751280

**Published:** 2026-05-21

**Authors:** Ze-Ying Wang, Qin-Bao Zhang, Xiao-Ming Zheng

**Affiliations:** 1 Department of Neurology, Hulunbuir People's Hospital, Hulunbuir, Inner Mongolia Autonomous Region, China; 2 Department of Cardiology, Hulunbuir People's Hospital, Hulunbuir, Inner Mongolia Autonomous Region, China

**Keywords:** acute ischemic stroke, comprehensive treatment, endovascular therapy, intravenous thrombolysis, medical quality, rehabilitation, secondary prevention

## Abstract

The leading cause of death and disability in Chinese residents is acute ischemic stroke (AIS). Standardized comprehensive treatment is essential for improving AIS outcomes. This article provides a systems-level analysis of the comprehensive treatment system of AIS in China by integrating evidence-based clinical guidelines, real-world clinical practice data and national medical quality control results, reviews the complete process from pre-hospital emergency care to ultra-early reperfusion therapy (intravenous thrombolysis and endovascular treatment) to the management of acute-phase complications to secondary prevention and rehabilitation. The article integrates the latest clinical research data, current diagnostic and treatment guidelines and medical quality control report in China, carries out a critical analysis on the strengths, weaknesses and regional imbalance of current treatment models by grading the evidence strength of different research types (randomized controlled trials, observational studies, guidelines and meta-analyses), and elaborates on future development direction which can serve as reference to improve overall treatment level of AIS in China.

## 1 Introduction

AIS has become a serious national public health problem due to its high incidence, disability rate, and mortality, placing a severe burden on society and families (1). According to the “China Stroke Prevention and Control Report 2024,” in 2021, there were 4.09 million new stroke cases, 26.34 million existing cases, and 2.59 million stroke deaths in China with the vast majority being ischemic stroke (1). The rising absolute case numbers due to an aging population offsets the benefits of treatment improvement (2), underscoring the necessity of controlling the increase in stroke burden associated with aging (2). In recent years, significant achievements have been observed in China's comprehensive therapy for AIS, and the accessibility of reperfusion therapy has greatly improved. As of the latest report, the number of stroke centers in China has reached 1,962 (1). Treatment strategies are being optimized and the rates of recurrence of stroke, disability and mortality are decreasing (3). Nevertheless, many challenges still exist in treatment today (4). Prehospital delay is the main reason behind low rates of reperfusion therapy application rate of reperfusion therapy is still suboptimal (4, 5). Disparities in the quality of healthcare exist by region and level of the hospital (6). Subsequently, the quality of treatment and standardization need to be improved.

This narrative review is designed as a systems-level analysis combined with an evidence-based therapeutic overview of AIS comprehensive treatment in China, which aims to clarify the current development status, clinical application dilemmas and future optimization directions of AIS treatment system in China by integrating global high-quality evidence with China-specific epidemiological, clinical and healthcare system data. This narrative review was based on a systematic literature search of PubMed, CNKI, Wanfang, Cochrane Library, and Web of Science databases for articles published between January 2010 and February 2026. The search strategy combined terms related to AIS, its management (e.g., intravenous thrombolysis, endovascular treatment, thrombectomy, stroke unit, secondary prevention, and rehabilitation), and the Chinese context. Two independent reviewers conducted literature screening, data extraction and quality assessment; any disagreements were resolved through consultation with the corresponding author. Inclusion criteria comprised: (1) studies conducted in China providing country-specific epidemiological or clinical data; (2) landmark global randomized controlled trials; (3) national and international clinical guidelines published between 2023 and 2026; and (4) systematic reviews and meta-analyses. Non-English/Chinese articles, basic experimental studies without clear clinical translational value, and studies with sample sizes < 100 were excluded. A total of 328 relevant literatures were initially screened, 189 were excluded due to inconsistent research themes or non-compliant study types, and 139 literatures were finally included for data synthesis and analysis, including 26 China-specific RCTs, 47 China-based observational studies, 16 clinical guidelines (including 7 Chinese national guidelines) and 50 global systematic reviews/meta-analyses. The synthesized evidence from this search forms the basis of this review, and the evidence strength of all cited studies is graded according to the Oxford Centre for Evidence-Based Medicine (OCEBM) 2011 Levels of Evidence criteria throughout the manuscript.

## 2 Current status of pre-hospital emergency care and stroke center construction

### 2.1 Public recognition and emergency response system

Raising public awareness about stroke recognition is crucial to improving prehospital delays. Research has shown insufficient public knowledge to be one of the causes of prehospital delays [(7), Level C evidence]. Despite some advancements in public education, this problem is especially acute in the remote regions, indicating the need for public awareness campaigns. An effective emergency response should facilitate instant conveyance of patients to hospitals capable of administering treatment. Though coverage and efficiency countrywide exist, there are major geographic disparities, and emergency transport is a key factor influencing access to endovascular therapy [(8), Level B evidence]. Optimizing conditions of emergency dispatch, making emergency services available and enforcing the policy to transfer the patient directly to the hospital that has the capacity are strategies that can reduce the loss of time during transfer [(7), Level C evidence]. How to achieve efficient coordination of emergency medical resources in the country? It is important to give priority to suspected stroke patients in the emergencies. Since 2006, prehospital time delays have been reduced by 27%. The proportion of patients transferred through emergency referrals has increased from 17% to 51% [(9), Level C evidence]. This advancement cannot be separated from standardization. Nonetheless, patients with milder signs and night-time onset are still at greater risk of tardy hospital admission [(9), Level C evidence]. As such, subsequent training must emphasize atypical symptoms detection and rapid response preparation.

### 2.2 Stroke center certification and regional collaborative treatment network

The country has promoted the establishment of a stroke center system of provinces, cities and counties, and the implementation of stroke center certification [(10), Level C evidence]. Since its establishment in 2015, stroke center certification in China has led to increased use of intravenous thrombolysis (IVT) with recombinant tissue-Type plasminogen activator (rt-PA) [(11), Level B evidence, China-based cohort study], which is an FDA-approved tissue plasminogen activator. Regional stroke care networks should achieve “initial diagnosis at primary care, two-way referrals and differentiated treatment for acute and chronic cases” by pursuing green channels for referrals and teleconsultation mechanisms [(12), Level B evidence]. Telemedicine has emerged as an effective tool to bridge urban-rural gaps, enabling remote stroke expertise to guide thrombolysis decisions in underserved areas [(12), Level B evidence]; however, its real-world applicability is limited by insufficient medical internet infrastructure in rural areas and the lack of standardized teleconsultation protocols in some regions. Essential performance metrics of stroke centers such as the door-to-needle time (DNT) have emerged as the focus of healthcare quality oversight [(13), Level A evidence, meta-analysis], but performance on these key measures differ by certification level [(13, 14), (13): Level A evidence; (14): Level B evidence]. As a result, ongoing monitoring and feedback mechanisms help stroke centers consistently revise their services as a whole to improve treatments.

## 3 The popularization and challenges of intravenous thrombolytic therapy

### 3.1 Current application status of alteplase IVT

The standard treatment of choice for AIS in the first 4.5 h of onset is IVT with rt-PA [(15), Level A evidence, global RCTs and guidelines]. In China, advanced stroke centers have been increasingly applying the procedure but national average thrombolysis rates remain low compared to developed countries. The primary obstacle is time delay, even though initiatives such as stroke green channels have reduced it in DNT. Mobile stroke units (MSUs) can reduce the time to treatment by transferring computerized tomography (CT) imaging and thrombolysis decision-making into the ambulance [(16), Level A evidence, RCT]. For certain patient groups thrombolysis decisions remain controversial. IVT is indicated for patients with minor stroke (NIHSS ≤ 5) [(15), Level A evidence]. Although it is considered safe and effective, a recent China-based RCT (ARAMIS trial [(17), Level A evidence]), showed that dual antiplatelet therapy was non-inferior to alteplase IVT in preventing stroke recurrence in minor non-disabling AIS patients, leading to the heterogeneity of clinical recommendations for this subgroup. For wake-up strokes or those with an obvious onset time, image-based selection for IVT in the extended window (4.5–9 h) is safe and effective [(18, 19), (18): Level B evidence; (19): Level A evidence, ESO guidelines]. In special populations like the elderly with functional independence, who may benefit from thrombolysis for better outcomes, the decision may be more complex [(20, 21), (20): Level B evidence, China-based cohort; (21): Level C evidence], due to the higher risk of intracranial hemorrhage in the elderly and the lack of large-sample RCTs focusing on Chinese elderly AIS patients. These controversies indicate that there's a requirement for better personalized risk assessment.

### 3.2 Novel thrombolytic drugs and exploration of extended time window

Genetically modified recombinant tissue-type plasminogen activator (TNK) is emerging as a novel thrombolytic agent in the setting of AIS because of its easy single intravenous bolus and longer plasma half-life. According to studies, TNK has more fibrin specificity than conventional rt-PA, which theoretically decreases the risk for systemic coagulation disorders and intracranial hemorrhage [(22), Level B evidence, systematic review]. The clinical studies have shown that TNK is either non-inferior or possibly superior to rt-PA in terms of safety and efficacy profile [(23, 24), (23): Level A evidence, systematic review; (24): Level C evidence, *in silico* study]; however these studies have notable heterogeneity in patient inclusion criteria (e.g., different age ranges and stroke severity) and TNK dosing regimens, leading to inconsistent conclusions regarding the superiority of TNK over rt-PA. Similar clinical outcomes with TNK are suggested by real-world studies [(25), Level C evidence, single-center study]. Due to its relatively low cost and ease of administration, TNK can be more advantageous [(25), Level C evidence] in resource-limited primary hospitals in China, where the technical threshold for drug administration is a key limiting factor for IVT application. According to the recommendations of the European Stroke Organisation and the American Heart Association/American Stroke Association, TNK is indicated for specific conditions [(26), Level A evidence, international guidelines]. An original multicenter phase III study evaluating the clinical efficacy and safety of TNK in comparison with alteplase is being done in China. It will furnish crucial evidence regarding the employment of TNK in Chinese patients [(27), Level B evidence, study protocol]. Nevertheless, the optimal TNK dose (0.25 mg/kg vs. 0.4 mg/kg) and its role in bridging therapy prior to endovascular thrombectomy remain subjects of ongoing investigation. The ORIGINAL trial is expected to clarify these issues in the Chinese population [(27), Level B evidence], and its results will fill the gap of high-quality evidence for TNK application in Chinese AIS patients, which is of great significance for the optimization of IVT regimens in China. These findings suggest that TNK may become a new option for thrombolytic therapy in AIS.

## 4 Rapid development and bottlenecks of endovascular therapy (mechanical thrombectomy)

### 4.1 Routine thrombectomy for large vessel occlusion and expansion of the time window

According to landmark global studies, such as the DAWN and DEFUSE-3 studies, endovascular thrombectomy (EVT) became the standard for anterior circulation large vessel occlusion (LVO) with Class I, Level A recommendation in major guidelines [(28, 29), Level A evidence, RCTs]. Moreover, its time window was expanded to 24 h after the onset of symptoms [(28, 29), Level A evidence]. Moreover, its time window was expanded to 24 h after symptom onset in patients selected by advanced imaging (e.g., perfusion-diffusion mismatch) [(28, 29), Level A evidence]. In comprehensive stroke centers, thrombectomy in late time window (6–24 h) patients selected with imaging has been routine, with improved procedure volumes and success rates [(30, 31), (30): Level B evidence; (31): Level C evidence]; however the strict imaging selection criteria mean only about 30% of late-onset LVO patients being eligible for EVT in real-world clinical practice in China. Nevertheless, the strict multimodal imaging selection is still difficult to implement in primary hospitals. Hence, simpler screening strategies such as non-contrast CT plus computed tomography angiography (CTA) have been studied to widen treatment availability [(32, 33), (32): Level C evidence; (33): Level A evidence, meta-analysis], and a recent China-based observational study confirmed that this simplified strategy has acceptable diagnostic accuracy for identifying eligible EVT patients in primary hospitals with limited imaging conditions. The findings regarding the effectiveness of thrombectomy in posterior circulation LVO are becoming more concrete, with several centers in China gaining experience with it. The optimal patient selection criteria and therapeutic window remain under investigation [(29, 34), (29): Level A evidence; (34): Level B evidence]. Three key RCTs (BASICS, ATTENTION, BAOCHE, [(29), Level A evidence]) have demonstrated the potential benefits of EVT for posterior circulation LVO, but there is still controversy about the optimal therapeutic window (6 h vs. 24 h) and imaging selection criteria for this subgroup, and the sample size of Chinese patients in these trials is relatively small, leading to limited evidence for Chinese clinical practice. The optimal patient selection criteria and therapeutic window remain under investigation [(29, 34), (29): Level A evidence; (34): Level B evidence]. Furthermore, advanced age should not be a barrier to EVT. Recent meta-analyses confirm consistent clinical benefit across all ages, including patients aged ≥80 years, who achieve comparable functional outcomes despite a marginally higher bleeding risk [(35), Level A evidence, meta-analysis]. These findings support individualized risk-benefit assessment rather than age-based exclusion for EVT in the elderly.

### 4.2 Thrombectomy techniques, devices, and perioperative management

As EVT becomes the standard of care for AIS due to LVOs, introduction of new thrombectomy techniques and devices and optimization of perioperative management can improve outcomes [(36), Level A evidence, systematic review]. Techniques including direct aspiration (e.g., the ADAPT technique) and stent retriever plus aspiration (e.g., the SWIM technique) have found widespread adoption. However, there is a lack of high-quality head-to-head studies to establish their absolute superiority [(37), Level B evidence, systematic review], and real-world data in China show that the choice of technique is mostly dependent on the operator's experience rather than standardized patient stratification, leading to inconsistent clinical outcomes. Second-generation thrombectomy devices (pRESET, EmboTrap, NeVa™) help improve recanalization rates [(38–40), (38): Level B evidence; (39): Level B evidence; (40): Level B evidence, all meta-analyses]; however the high cost of these devices limits their popularization in primary and secondary hospitals in China, which is a major barrier to the equitable access of EVT. The prognosis of the patient is significantly influenced by their perioperative management involving blood pressure control and anesthesia selection [(41, 42), (41): Level B evidence; (42): Level C evidence]. The collaborative management processes that are a standard and multidisciplinary in nature are of decisive significance for maximizing the benefits of EVT [(43, 44), (43): Level C evidence; (44): Level B evidence]; however the lack of standardized perioperative management protocols and insufficient multidisciplinary collaboration in some regional stroke centers lead to suboptimal EVT outcomes in China's real-world practice.

## 5 Prevention and management of acute phase complications and critical care

### 5.1 Management of cerebral edema and malignant intracranial hypertension

Lethal malignant cerebral edema due to large-area cerebral infarction is a cause of early death of patients and decompressive craniectomy (DC) is a useful life-saving surgical technique. Studies show that early DC may lower ICP and prevent brain herniation in patients with malignant middle cerebral artery infarction due to LVO in the anterior circulation [(45), Level B evidence, scoping review]. Emergency DC provides a survival opportunity for patients suffering from malignant cerebral venous sinus thrombosis (CVST) [(46), Level C evidence, China-based case series]. The timing of operation is very critical meaning patients developing malignant edema after EVT may require DC earlier [(47), Level B evidence]. Ultrasonic assessment of the optic nerve sheath diameter (ONSD) can help detect severe intracranial hypertension [(48), Level B evidence]. The controlling of ICP through medication is significant medically. However, excessive use of medications for ICP may cause complications including acute kidney injury [(49), Level B evidence]. Oral glibenclamide and other exploratory treatments targeting the mechanisms of cerebral edema require a risk–benefit assessment [(50), Level C evidence], as current small-sample studies show inconsistent efficacy and potential hypoglycemic side effects, and there is no high-quality evidence for their application in Chinese AIS patients. An improved understanding of the relationship between glymphatic system dysfunction and edema presents new insights for future therapies [(51), Level C evidence, basic study].

### 5.2 Hemorrhagic transformation and infectious complications

The risk of symptomatic hemorrhagic transformation after the use of reperfusion therapy for AIS is not uncommon and may happen due to blood-brain barrier disruption, reperfusion injury, and patient characteristics [(52), Level B evidence, systematic review]. Risk factors for poor outcomes include old age, high blood pressure, high blood sugar level and the size of the stroke [(53, 54), (53): Level C evidence; (54): Level B evidence, China-based retrospective study]. Elevated serum levels of follistatin-like protein 1 and matrix metalloproteinase-9 and imaging parameters increased relative cerebral blood flow values, are also predictors of haemorrhagic risk [(55, 56), (55): Level C evidence; (56): Level B evidence]. The stress-induced hyperglycemia ratio is independently associated with early neurological deterioration after IVT [(57), Level B evidence, China-based cohort study]. The use of personalized prevention and control measures requires a clinical assessment. Infections after stroke, particularly pneumonia and urinary tract infections, impede recovery. Their mechanism is post-stroke immune suppression [(58), Level B evidence]. Being old, having a coma and having diabetes by itself is risk factors for hospital-acquired infection [(59), Level B evidence]. Infection rates can be diminished via the bundle approach like hand hygiene, early mobilization [(60), Level B evidence]. Intravenous thrombolytic is associated with a lower risk of complications [(60), Level B evidence]. Infection is related to poor outcomes and higher mortality [(58, 60), Level B evidence]. To reduce the harm caused by infections, stroke units should incorporate early screening and preventative care; however the implementation of these preventive measures in primary hospitals in China is limited by insufficient nursing resources and non-standardized infection control protocols.

## 6 Initiation and optimization of early secondary prevention

### 6.1 Antiplatelet and anticoagulation treatment strategies

In the case of non-cardioembolic stroke, the standard short-term regimen is dual therapy with aspirin plus clopidogrel which effectively lowers the risk of early recurrence of stroke [(61), Level A evidence, guidelines and systematic review]. Nonetheless, treatment for a duration of greater than 3 months might increase the bleeding risk without any added benefits [(62), Level B evidence]. Therefore, guidelines recommend a switch to single-agent long-term prevention after short-term therapy [(63), Level A evidence, guidelines]. The effectiveness of clopidogrel is affected by polymorphisms in the CYP2C19 gene [(61), Level A evidence] and genetic testing can be beneficial [(64), Level B evidence, China-based genetic study]; however genetic testing is not widely available in primary hospitals in China due to cost and technical limitations, leading to the blind use of clopidogrel in most clinical settings. Direct oral anticoagulants (DOACs) are the drugs of choice for cardioembolic stroke and are safer than warfarin [(61), Level A evidence] and improve outcomes while decreasing bleeding risk [(65), Level B evidence, China-based cohort study]. It is necessary to assess the characteristics of the stroke, risk of bleeding and the patient, and the choice of DOACs in China is also affected by drug affordability and insurance coverage, which limits the equitable access of optimal anticoagulant therapy for cardioembolic stroke patients in rural and low-income areas.

### 6.2 Timing principles for carotid revascularization (CEA/CAS)

While antiplatelet and anticoagulant therapies form the cornerstone of medical management, carotid revascularization, including Carotid Endarterectomy (CEA) and Carotid Artery Stenting (CAS), is critical for preventing recurrent stroke in patients with significant carotid stenosis. However, the timing of these interventions in the context of AIS requires careful stratification to balance the risk of early recurrence against the risk of hemorrhagic transformation.

For patients with AIS complicated by severe ipsilateral carotid stenosis (≥70%), current Chinese guidelines and clinical practices generally recommend deferring revascularization procedures until at least 2 weeks after stroke onset, with extended waiting in selected cases [(66), Level A evidence]. Performing CEA or CAS during the hyperacute phase is associated with an elevated risk of hemorrhagic transformation due to disrupted blood-brain barrier integrity and reperfusion injury. Delaying the intervention allows for the stabilization of the infarcted tissue and reduces perioperative bleeding complications. For patients with symptomatic carotid stenosis (e.g., those experiencing recent TIA or minor AIS who have stabilized), revascularization should be evaluated once the patient's neurological status is stable, ideally within 2 weeks of the index event [(67), Level A evidence, Chinese guidelines], which is slightly different from the American Heart Association (AHA) guidelines that recommend individualized assessment for revascularization at 48 h to 2 weeks after stroke onset for stable patients [(68), Level B evidence]. This discrepancy primarily stems from differences in baseline patient characteristics, variations in medical resource allocation, and the lack of a standardized postoperative management system for hyperacute interventions in many Chinese hospitals. Conversely, for patients with asymptomatic severe stenosis, the timing of surgery should be individualized, weighing potential long-term benefits against immediate risks based on the patient's age and comorbidities [(66, 67), Level A evidence, Chinese guidelines].

Special attention is required for the elderly population. Evidence suggests distinct differences in efficacy and safety profiles between CEA and CAS in older adults [(69), Level A evidence, meta-analysis]. While CEA has historically been the gold standard, CAS may carry a potentially higher risk of periprocedural embolic events in the elderly, particularly in those over 70 years of age [(69), Level A evidence]. Therefore, perioperative management for this demographic must focus on rigorous blood pressure control, antiplatelet optimization, and close monitoring for hyperperfusion syndrome to mitigate stroke risks, and Chinese clinical practice lacks standardized perioperative management protocols for geriatric patients undergoing carotid revascularization, leading to inconsistent clinical outcomes. Integrating these timing principles into clinical pathways ensures that revascularization therapy delivers maximal net benefit for AIS patients in China; however the implementation of these principles is limited by the uneven distribution of vascular surgery resources in China, with most primary hospitals lacking the capability to perform CEA/CAS.

### 6.3 Comprehensive control of risk factors

The secondary prevention of AIS focuses on the management of risk factors in order to stabilize, prevent, and improve outcome. Administering a high dose statin when the patient presents with a coronary event during the acute phase is one of the mainstays. This is because it not only lowers low-density lipoprotein cholesterol (LDL-C) but also stabilizes plaques, exerts anti-inflammatory effect, and improves endothelial function. Ultimately, it lowers the risk of a recurrence [(63), Level A evidence]. Present Chinese and international guidelines recommend to having very strict low-density lipoprotein cholesterol (LDL-C) targets (e.g., < 1.8 mmol/L or ≥50% reduction) specifically for high-risk patients [(70), Level A evidence, guidelines]; however real-world data in China show that only about 40% of AIS patients achieve the recommended LDL-C target due to poor medication adherence and insufficient follow-up. Both blood pressure and blood glucose management stress stable control without excessive fluctuations [(63), Level A evidence]. More than 85% of stroke risk is attributable to modifiable factors which highlights the importance of comprehensive control [(63), Level A evidence]. In addition to the conventional “three highs” (hypertension, hyperlipidemia, and hyperglycemia), atrial fibrillation (AF) is a risk factor for cardioembolic events. Anticoagulation strategies should be very thoughtful in these patients [(71), Level B evidence]. Anemia can indirectly cause stroke by diminishing the oxygen-carrying capacity of the blood which in turn affects cerebral perfusion, necessitating prompt management [(72), Level C evidence, case report]. Comprehensive management includes an effective risk assessment of lifestyle problems such as smoking and obesity to execute a customized whole-process care process with the aim to reduce rates of recurrence, disability and mortality [(73), Level A evidence, 2026 clinical guideline], and the main challenge in China is the lack of a perfect community-based follow-up system, leading to poor long-term adherence to risk factor control measures in AIS patients.

.

## 7 Early rehabilitation and multidisciplinary collaborative model

### 7.1 Timing and content of early rehabilitation intervention

The scope of “early rehabilitation” in the management of AIS is encouraging, which refers to the early admission of a patient after stabilization of vital signs usually within the next 24–48 h after onset [(74), Level B evidence]. A nationwide study conducted in Japan shows that the majority of stroke centers implement passive movement on the first day of hospitalization and commence out of bed activities on the second day [(74), Level B evidence, Japan-based nationwide study], and similar practices are adopted in most advanced stroke centers in China, but primary hospitals often delay the initiation of early rehabilitation due to insufficient rehabilitation therapists. The purpose of early rehabilitation is to prevent complications and take advantage of the “sensitive window period” of neuroplasticity to elicit functional recovery [(75), Level B evidence]. According to studies, early rehabilitation has a very strong association with improved activities of daily living of patients [(76), Level B evidence]. A large cohort study confirmed that starting rehab within 3 days of hospitalization is independently associated with better functional outcomes [(76), Level B evidence]. Additionally, another key factor regarding rehabilitation is the frequency and intensity of rehabilitation sessions, with a higher frequency producing superior functional outcomes at discharge [(77), Level B evidence]. Rehabilitation programs now include a full system that incorporates swallowing and speech therapy, psychology, and rehabilitation nursing [(78), Level B evidence]. New technologies involving robot-assisted gait training show advantages for physical therapy [(79), Level B evidence]. Improvement in function through early comprehensive oral care and neuromuscular electrical stimulation for dysphagia [(80), Level B evidence, pilot RCT]. Psychological interventions are unavoidable, and the use of virtual reality training in early rehabilitation is additionally found more effective in reducing depression and anxiety [(81, 82), (81): Level B evidence; (82): Level B evidence]. Early use of rehabilitation nursing is superior to traditional care in improving neurological, motor and activities of daily living function [(83), Level C evidence]. Nutritional support is an important aspect too. L-carnitine supplementation improves malnutrition status early on [(84), Level A evidence, RCT]. In conclusion, a modern early rehabilitation for AIS is a program that is organized, comprehensive multidisciplinary treatment that begins in the ultra-early phase with the aim of optimizing functional recovery and quality of life of patients, and the main challenge in China is the uneven distribution of rehabilitation resources, with rural and remote areas lacking professional rehabilitation personnel and standardized rehabilitation programs.

### 7.2 Stroke unit and multidisciplinary team development

The stroke unit, as the core organizational form of comprehensive treatment for AIS, derives its essential value from providing integrated and standardized diagnosis and treatment services to patients through substantive multidisciplinary team collaboration [Level A evidence, guidelines and meta-analyses]. Although advanced stroke centers have been widely established domestically, there are significant disparities in their actual operational quality, with the key factor being whether multidisciplinary team collaboration has truly achieved normalization and high efficiency [Level C evidence, China-based observational data]. Research indicates that substantive multidisciplinary team collaboration can significantly improve patient outcomes. For instance, after implementing multidisciplinary team interventions in stroke units, the time for patients with aneurysmal subarachnoid hemorrhage to initiate sitting, standing, and walking was notably reduced, along with decreased hospitalization duration [(85), Level C evidence]. In resource-limited settings, establishing stroke units and adhering to the basic framework of multidisciplinary collaboration can also improve patient outcomes [(86), Level C evidence]. Key elements of substantive collaboration include regular, structured multidisciplinary ward rounds. The use of standardized stroke-specific ward round documentation forms can significantly enhance the quality and consistency of documentation [(86), Level C evidence]. In a UK study, the introduction of structured daily stroke ward round documentation forms improved adherence to recording key components [(87), Level B evidence]. Additionally, multidisciplinary teams play a crucial role in managing complications such as post-stroke dysphagia [(88), Level B evidence]. Therefore, the effective operation of stroke units relies on substantive team building, where multidisciplinary members break down barriers, communicate regularly, and make joint decisions under the leadership of neurology departments, and in China, the main bottleneck is that most primary stroke centers only have a formal multidisciplinary team structure but lack regular and standardized collaborative mechanisms, leading to a failure to realize the full value of stroke units.

## 8 Medical quality improvement and future prospects

### 8.1 Quality control metrics and continuous improvement

In China, the national stroke quality control program has established a set of performance indicators, including door-to-needle time, door-to-puncture time, recanalization rate, and symptomatic intracranial hemorrhage rate. Data from the China Stroke Center Alliance indicate substantial improvements in these metrics over the past decade; yet significant disparities persist across hospitals of different certification levels and geographic regions [(6, 13, 14), (6): Level B evidence, China-based nationwide study; (13): Level A evidence; (14): Level B evidence]. These disparities are fundamentally driven by an uneven distribution of medical resources, as many primary hospitals still lack advanced imaging equipment, professional stroke care personnel, and standardized treatment workflows, with urban comprehensive stroke centers having significantly better performance in key quality metrics than rural primary stroke centers (e.g., average DNT: 58 min vs. 89 min). Continuous quality improvement initiatives, such as the “Stroke Quality Control and Improvement Program,” have been implemented to standardize care delivery [Level C evidence, national health policy]. Real-time data feedback, case-based training, and regional peer comparison have been shown to effectively reduce treatment delays and improve clinical outcomes [Level B evidence]. Ongoing efforts should focus on narrowing urban-rural gaps and expanding quality monitoring to secondary prevention and long-term rehabilitation, and the key to achieving this goal is to increase investment in primary medical institutions and establish a standardized regional stroke quality control network.

### 8.2 Future development directions

It is crucial to promote MSUs to reduce onset-to-treatment time by carrying out CT scans and thrombolytic therapy in the ambulance for future AIS care [(89–91), (89): Level B evidence; (90): Level C evidence, China-based provincial data; (91): Level B evidence]. In China, the MSUs has special significance in improving the efficiency of stroke emergencies in remote or congested areas of cities [(91), Level B evidence]; however the promotion of MSUs in China is limited by high construction and operation costs, and only a few large cities (e.g., Beijing, Shanghai, and Guangzhou) have established MSU systems so far. Telemedicine networks are also expected to expand, facilitating remote stroke expertise to primary care centers and reducing geographic disparities in reperfusion therapy access [(12), Level B evidence], and the future optimization direction is to combine telemedicine with artificial intelligence to develop an intelligent stroke diagnosis and treatment system suitable for primary medical institutions in China. Artificial intelligence (AI) tools that can interpret images, predict outcome and guide rehabilitation are revolutionizing diagnosis and treatment. The imaging data can be processed quickly with the help of AI, which also aids LVO [(92), Level B evidence] identification and facilitates extending the treatment time window [(91), Level B evidence], and several China-based AI research projects have achieved promising results in stroke imaging interpretation, but the clinical translation and large-scale application still face challenges such as lack of unified technical standards and clinical validation. Improving the long-term quality of life of our patients will also require strengthening basic and clinical translational research, exploring new therapies such as neuroprotection and brain cell regeneration, and enhancing the full life-cycle health management chain from emergency care to community-based management [(93–95), (93): Level B evidence; (94): Level B evidence; (95): Level B evidence], and in China, it is necessary to establish a closer collaboration between basic medical research institutions and clinical hospitals to accelerate the translational application of new AIS therapies, and build a perfect community-based follow-up and management system to realize the full life-cycle care of AIS patients.

## 9 Conclusion

In China, a comprehensive treatment system for AIS was first established, and its treatment capabilities were enhanced by the establishment of the stroke center network and the use of thrombolysis and interventional techniques. This has been made possible by advancement in medical technology, public health policies, integration of resources. Based on the critical analysis of 139 included literatures with graded evidence strength, the current AIS treatment system in China still faces prominent challenges including pre-hospital delays, unequal regional standards for diagnosis and treatment, limited real-world applicability of advanced medical technology due to cost and resource constraints, and weaknesses in long-term follow-up and management. The productivity of the system is still challenged by the presence of pre-hospital delays, unequal regional standards for diagnosis and treatment, embeddedness of fitting technology and long-term management weakness. In the future, the focus must be on quality, equity, and sustainable development. To build a high-quality system, we must further strengthen evidence-based clinical pathways by grading and applying high-quality evidence, promote uniform diagnosis and treatment standards across different levels of hospitals, enhance primary care capabilities through resource investment and technical training, integrate traditional Chinese medicine and other treatment modules based on scientific evidence, and implement standardized multidisciplinary cooperation and early rehabilitation protocols. Going forward, a systems-thinking approach needs to be adopted to achieve balance between several strategies, realize the shift from the construction of the stroke treatment system to efficiency improvement and equitable access, and thus reduce the disease burden effectively. The future development of AIS treatment in China should focus on solving the practical problems in real-world clinical practice, such as optimizing the cost-effectiveness of advanced therapies, establishing a standardized regional medical collaboration network, and building a perfect full life-cycle health management chain, so as to continuously improve the overall level of AIS comprehensive treatment and reduce the incidence, disability rate and mortality of AIS in China.
